# The association between a pro‐inflammatory diet and machine learning‐based brain age in middle‐aged and older adults: Findings from the UK Biobank

**DOI:** 10.1002/alz.086979

**Published:** 2025-01-09

**Authors:** Michelle M. Dunk, Huijie Huang, Jiao Wang, Abigail Dove, Sakura Sakakibara, Jie Guo, Adrián Carballo‐Casla, David A. Bennett, Weili Xu

**Affiliations:** ^1^ Aging Research Center, Karolinska Institutet, Stockholm Sweden; ^2^ School of Public Health, Tianjin Medical University, Tianjin China; ^3^ Army Medical University, Chongqing, Chongqing China; ^4^ Aging Research Center, Karolinska Institutet, Stockholm, Stockholm Sweden; ^5^ China Agricultural University, Beijing, Beijing China; ^6^ CIBER of Epidemiology and Public Health (CIBERESP), Madrid Spain; ^7^ Rush Alzheimer’s Disease Center, Rush University Medical Center, Chicago, IL USA

## Abstract

**Background:**

Pro‐inflammatory diets have been associated with cognitive decline and dementia, but their impact on overall brain aging is unclear. We aimed to investigate the relationship between a pro‐inflammatory diet and a machine learning‐based measure of brain age, taking into account age, genetic risk for Alzheimer’s disease (AD), and systemic inflammation.

**Method:**

From the UK Biobank, 21,473 participants aged 40‐70 years and free of chronic brain diseases were included. Baseline Dietary Inflammatory Index (DII) scores were calculated from participants’ average intake of 31 nutrients, assessed up to five times between 2009‐2012 using the Oxford WebQ. Participants were categorized into DII tertiles (low [anti‐inflammatory], moderate, or high [pro‐inflammatory]). An AD polygenic risk score (PRS_AD_; tertiled as low, moderate, and high) and high‐sensitivity C‐reactive protein (hsCRP; a biomarker of systemic inflammation) were measured from baseline blood draw. We used least absolute shrinkage and selection operator (LASSO) regression to estimate brain age from 1,079 structural and functional magnetic resonance imaging (MRI) measures, obtained approximately 9 years after baseline. We calculated brain‐predicted age difference (BPAD; the difference between brain age and chronological age), where BPAD >0 indicates accelerated brain aging. Data were analyzed using linear regression and mediation analysis.

**Result:**

Among all participants, DII scores ranged from ‐6.22 to 5.38 (low: ‐6.22 to ‐1.34; moderate: ‐1.35 to 0.49; high: 0.50 to 5.38). In multi‐adjusted linear regression, each unit increase in DII score was associated with older brain age by β = 0.07 (95% confidence interval: 0.02, 0.12) years and greater BPAD by 0.06 (0.02, 0.11) years. Compared to the low DII tertile, high DII was associated with older brain age by 0.35 (0.12, 0.58) years and greater BPAD by 0.34 (0.13, 0.55) years. These associations were pronounced in adults aged ≥60 years, attenuated in adults aged 40‐59 years, and did not differ by PRS_AD_. HsCRP levels mediated 10% of the DII‐brain age association and 14% of the DII‐BPAD association.

**Conclusion:**

A pro‐inflammatory diet is associated with accelerated brain aging, especially in older adults and independently of genetic risk for AD. Systemic inflammation may partially mediate the association between a pro‐inflammatory diet and brain age.

